# Impact of a dedicated antenatal specialist service for women with a history of stillbirth: the rainbow clinic

**DOI:** 10.1186/s12884-025-07421-6

**Published:** 2025-03-19

**Authors:** Jack Le Vance, Michelle Plant, Samiramis Saba, Alexander E.P. Heazell, R. Katie Morris, Victoria Hodgetts Morton, Leo Gurney

**Affiliations:** 1 Institute of Applied Health Research, University of Birmingham, Birmingham, B15 2TT UK; 2 Birmingham Women’s and Children’s NHS Foundation Trust, Birmingham, B15 2TG UK; 3 Maternal and Fetal Health Research Centre, University of Manchester, Manchester, M13 9PL UK; 4 Saint Mary’s Hospital, Manchester University NHS Foundation Trust, Manchester, M13 9WL UK

**Keywords:** Antenatal care, Fetal outcomes, Maternal outcomes, Stillbirth, Rainbow clinic

## Abstract

**Background:**

Stillbirth in a prior pregnancy represents a significant risk factor for a subsequent stillbirth and other adverse pregnancy outcomes. There is a developing body of evidence supporting the implementation of dedicated specialist antenatal service provision for women with a history of stillbirth in future pregnancies. We aimed to assess the impact of our specialist service, the Rainbow Clinic, on maternal-fetal outcomes.

**Methods:**

A retrospective case-control study was conducted comparing women with a history of previous stillbirth prior to and following the inception of the Rainbow Clinic at the Birmingham Women’s Hospital, United Kingdom. Case records were reviewed from 2017 to August 2024. The Rainbow service was implemented on 9th May 2022; therefore, this became our cut off to define case and control groups. The control group matched the Rainbow Clinic eligibility criteria. Individual maternal and fetal outcome data were collected from case records. A composite adverse perinatal outcome was defined as one of: perinatal mortality; an Apgar score < 7 at five minutes or an umbilical artery pH < 7.05, or both; admission to NICU; intraventricular hemorrhage; hypoxic ischemic encephalopathy; necrotizing enterocolitis; retinopathy of prematurity; respiratory distress syndrome; pneumonia; and neonatal sepsis.

**Results:**

Eighty-seven women were seen after establishment of the Rainbow Clinic group compared with 65 women in the pre-Rainbow Clinic control group. 91% of the Rainbow Clinic group were prescribed aspirin compared to 70% within the pre-Rainbow Clinic group (*p* = 0.001). The rate of composite adverse perinatal outcome was significantly less in the Rainbow versus the pre-Rainbow Clinic group (Odds Ratio (OR), 0.46 [95% Confidence Interval (CI), 0.22–0.98]). Women in the Rainbow Clinic were statistically more likely to have a prelabor cesarean birth (OR 2.44 [95% CI, 1.20–4.94]), however, gestational age at delivery was significantly greater within the Rainbow Clinic group (median 38 weeks 0 days versus 37 weeks 3 days, *p* = 0.004), including a significant reduction in cases of very and extreme preterm delivery (OR 0.17 [95% CI, 0.03–0.80] and OR 0.05 [95% CI, 0.00–0.93] respectively). 8% of the pre-Rainbow Clinic group had a further stillbirth or second trimester miscarriage compared to 2% within the Rainbow Clinic group (*p* = 0.07).

**Conclusion:**

This study provides data on the beneficial impact of a specialist pregnancy after loss service on clinical outcomes. Continued research, including qualitative analysis of this service is necessitated to determine the efficacy of these specialist clinics.

## Introduction

In the United Kingdom (UK), stillbirth is defined as a baby that is born without signs of life after 24 weeks’ gestation [1]. In recent years, stillbirth rates within the UK have declined due to national initiatives such as the National Health Service (NHS) England Saving Mothers and Babies Lives Care Bundles in response to nationally implemented ambitious targets [2, 3]. However, with a current stillbirth rate of 3.35 per every 1000 total births, this figure remains higher than other comparable high-income countries [1]. Furthermore, there is a considerable disparity in stillbirth rates across the UK, with the West Midlands having one of the highest rates at 4.3 per 1000 total births, whilst the South-West displaying rates of 2.9 stillbirths per 1000 total births [4]. Disparities in reported figures suggest there is still a considerable opportunity to reduce stillbirths further.

Stillbirth in a prior pregnancy represents a significant risk factor for a subsequent stillbirth. A large systematic review of 16 studies comprising 3,412,079 pregnancies demonstrated a 2.5% risk of stillbirth in women with a previous stillbirth, compared to 0.4% in those with no such history (pooled odds ratio (OR) 4.8, 95% confidence interval (CI) 3.77–6.18) [5]. Additionally, women with a previous stillbirth are at increased risk of additional antenatal and intrapartum complications, such as pre-eclampsia (OR 3.1, 95% CI 1.7–5.7), low birthweight (OR 2.8, 95% CI 1.7–4.5), preterm delivery (OR 2.8, 95% CI 1.9–4.2) and emergency cesarean section (OR 2.1, 95% CI 1.5–3.0) [6]. Importantly, subsequent pregnancies following an index stillbirth can be a hugely distressing experience and psychologically impactful, both for the women and family [7–9]. This can be compounded by a lack of continuity with clinicians and antenatal appointments, leading to challenges in patient communication and the feeling of reduced psychological support [10, 11].

Consequently, in light of the risk factors posed to this cohort of women within subsequent pregnancies, alongside further guidance set out by the Royal College of Obstetricians and Gynecologists, several hospitals have begun to develop specialist antenatal services termed Rainbow Clinics [12, 13]. However, it should be noted that there is still a wide variation in the care provided in pregnancies after stillbirth, which was clearly represented in a recent online survey of 138 UK maternity units [14].

The Rainbow Clinic at the Birmingham Women’s Hospital, UK was established in May 2022. This clinic aimed to provide specialist antenatal care and psychological support for women with a history of pregnancy loss. Prior models of this multidisciplinary service within alternative maternity units have demonstrated improved maternal-fetal outcomes, particularly in relation to reduced prematurity and low birthweight [15]. Therefore, following two years since clinic inception, we aimed to analyze the impact of our Rainbow Clinic service on maternal and neonatal outcomes.

## Materials and methods

We conducted a retrospective case-control study by reviewing the maternal and fetal clinical outcomes of women with a previous history of stillbirth prior to the inception of the Rainbow Clinic service with those who attended the newly established clinic. Electronic maternity records were used to identify all women who had a subsequent pregnancy at the Birmingham Women’s hospital following a prior stillbirth at any prior time. Case records were reviewed from 2017 to August 2024. The Rainbow service was implemented on 9th May 2022; therefore, this became our cut off to define case and control groups. The study period spanned over seven years to ensure the study team could acquire a suitable sample size within both the control and case groups to enable comparisons to be made. No formal sample size calculation was performed for this study, but was determined pragmatically.

Women with a singleton pregnancy were eligible for inclusion within the Rainbow Clinic service if they had previously had a stillbirth following 24 weeks’ gestation or an early neonatal death with unexplained or likely placental cause. Women with a prior stillbirth secondary to prematurity, congenital or genetic abnormalities were not eligible for the Rainbow Clinic and were seen in their respective specialist antenatal clinics. In certain cases, women with late second trimester losses, not secondary to prematurity but likely related to a placental cause were eligible for review within the Rainbow Clinic. Women with complex maternal or fetal medical conditions were recommended to be seen within their respective specialist antenatal clinic. The control group was matched to the inclusion criteria for the Rainbow Clinic to enable equal comparisons to be made between cohorts with respect to outcomes.

**Fig. 1:**
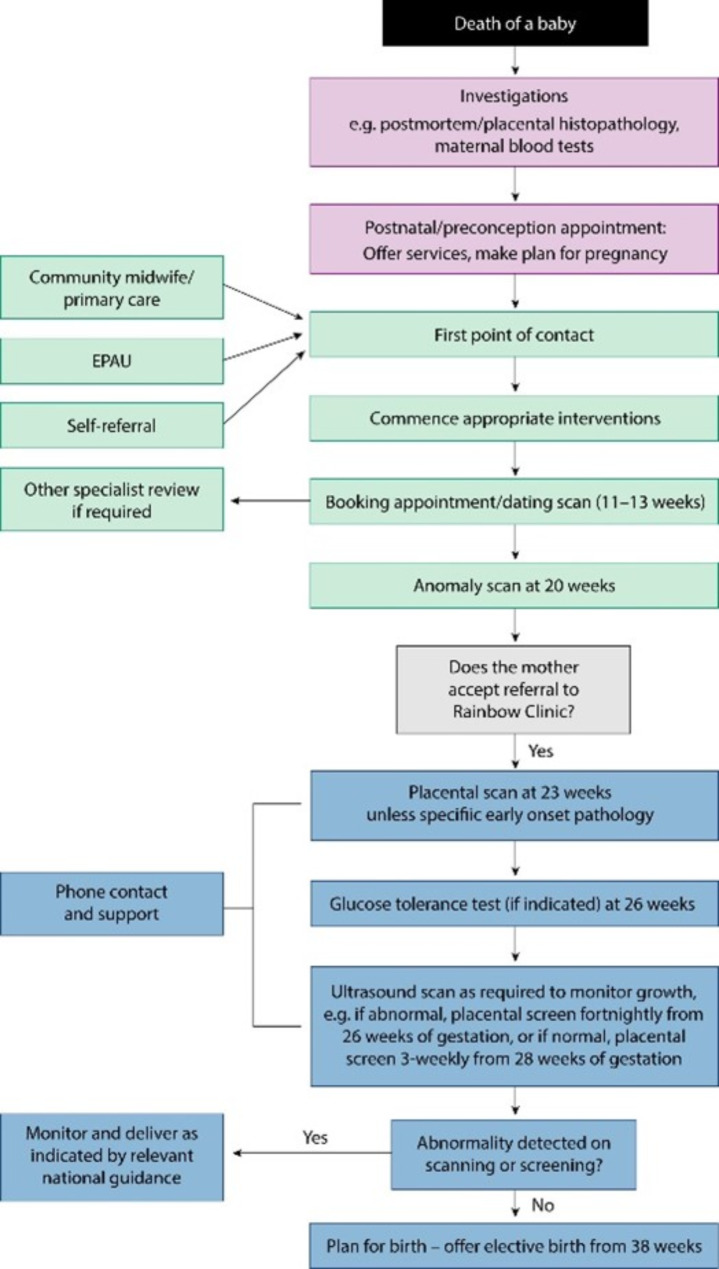
Care pathway for women with a previous history of stillbirth. EPAU: early pregnancy assessment unit. Reproduced from Graham N, Stephens L, Heazell AEP. Care in pregnancies subsequent to stillbirth or perinatal death. TOG. 2020; 23: 48–59 [12]. Reproduced under the Creative Commons Attribution License

The pathway of care for the Rainbow Clinic is based on the established service situated at Saint Mary’s Hospital in Manchester, which has been continuing since 2013 (Fig. 1) [12]. Care is established following assessment of index stillbirth cause, with review of the postmortem findings. Subsequent pregnancy management is based on prior cause, for example, women with evidence of placental insufficiency will receive Aspirin from an early viability scan. Following completion of the dating scan and mid trimester anomaly scan, cases meeting the criteria for referral are then reviewed at 23 weeks within the Rainbow Clinic for a placental assessment, including uterine artery Doppler velocimetry [16]. This entails a caliper measurement of the placental length and thickness to generate a placental ratio. A ratio cut off < 40 or > 40 will determine the timing of serial ultrasound assessment. A placental ratio < 40 necessities fortnightly growth scans from 26 weeks gestation. A placental radio > 40 will receive three to four-weekly serial growth scans from 28 weeks gestation. Gestational age to recommend delivery is then based on a variety of factors including the trajectory of fetal growth and development of any maternal-fetal antenatal complications. However, if the growth is linear and minimal complications arise antenatally, the recommendation for delivery is at 38 weeks’ gestation. The patient will be reviewed by the same dedicated clinical and midwifery team from 23 weeks until delivery, ensuring continuity of care [12].

Demographic data were collected for each group including: age, ethnicity, gravidity, parity, body mass index (BMI) smoking status, aspirin usage, low molecular weight heparin usage, number of antenatal clinician reviews and number of fetal ultrasound scans. Maternal outcome data collected included: development of hypertensive disease (gestational hypertension or pre-eclampsia), onset of intrapartum care (spontaneous, induction of labor, prelabor cesarean section), mode of birth (vaginal delivery, instrumental delivery, elective or emergency cesarean section (prelabour and intrapartum)), blood loss following delivery, maternal sepsis and prolonged postnatal admission > 5 days.

Fetal outcome data included: sex, birthweight, gestational age at birth, outcome (livebirth, stillbirth or second trimester miscarriage), Apgar scores at one and five minutes, umbilical artery cord pH, admission to the neonatal intensive care unit (NICU), duration of NICU stay and development of neonatal complications. Moderately premature, very premature and extreme prematurity were defined as gestational ages at delivery of < 37 weeks, < 32 weeks and < 28 weeks’ gestation respectively. Low birthweight was defined as < 2500 g at delivery. Birthweight centiles were grouped to < 10th centile, 10th -90th centile and > 90th centile. Customized growth charts were used in both cohorts. From 2017 to February 2019 customized charts were produced by K2 medical systems. From February 2019 onwards, customized GROW charts, produced by the Perinatal Institute were used.

Adverse perinatal outcomes were additionally defined as a composite outcome given the small frequencies of individual occurrences. Development of our composite outcome was informed by the recently published core outcome set for stillbirth, whilst pragmatically incorporating outcome data that was routinely recorded and widely accessible within the electronic maternity records [17]. The composite outcome consisted of any of the following: perinatal mortality (second trimester loss, stillbirth or neonatal death); an Apgar score below 7 at 5 min or an umbilical artery pH below 7.05, or both; admission to NICU; intraventricular hemorrhage; hypoxic ischemic encephalopathy; necrotizing enterocolitis; retinopathy of prematurity; respiratory distress syndrome; pneumonia; and neonatal sepsis.

All raw data were reported as frequencies with either mean and standard deviation (SD) or median with interquartile range. Differences in the demographic data, maternal complications, fetal and maternal clinical outcomes between those seen within and prior to the Rainbow Clinic were assessed using the students t-test for continuous variables or the X^2^ for categorical variables. When expected counts in either group were fewer than 5, the Fisher exact test was applied. Dichotomous outcome data were assessed with 2 × 2 tables constructed to calculate odds ratio (OR) and 95% confidence intervals (CI). Statistical analysis was performed using GraphPad PRISM 7.05 (GraphPad Software, La Jolla, CA, USA). A p value < 0.05 was considered statistically significant.

## Results

Eighty-seven women were reviewed within the specialist Rainbow Clinic and have subsequently given birth since its inception in May 2022. Sixty-eight women had a history of a single stillbirth, six women had a single neonatal death, four women had a single second trimester loss, four women had both an early neonatal loss and stillbirth, two women had a prior stillbirth and additional second trimester loss, two women had two prior stillbirths, and one woman had a history of a neonatal death, stillbirth and second trimester loss. Sixty-four women were included within the pre-Rainbow Clinic group, whereby 62 women had a prior history of single stillbirth, one woman had two prior stillbirths, and one woman had a stillbirth and a second trimester loss. The demographic characteristics of each group can be seen within Table 1. Women within the Rainbow Clinic group were statistically more likely to be commenced on aspirin (OR, 4.17 [95% CI, 1.69–10.29]). There were no other differences in all other aspects of the demographic data.

**Table 1:** Demographic characteristics of women in the rainbow cohort and pre-Rainbow cohort

			Rainbow cohort (*n* = 87)	Pre-Rainbow Cohort (*n* = 64)	*P* Value
Age (years; mean (SD))			32.0 (5.3)	30.6 (5.6)	0.12
Gravida (median (IQR))			3 (3–5)	3 (3–4)	0.68
Parity (median (IQR))			2 (1–3)	2 (1–3)	0.90
BMI (kg/m^2^; mean (SD))			28.3 (6.1)	27.7 (5.9)	0.57
Ethnicity n (%)					
	White British		32 (37%)	26 (41%)	0.63
	White European		3 (3%)	6 (9%)	0.17
	Asian		40 (46%)	25 (39%)	0.40
	African		7 (8%)	4 (6%)	0.76
	Caribbean		5 (6%)	3 (5%)	1.00
Current smoker n (%)			3 (3%)	7 (11%)	0.10
Aspirin in pregnancy n (%)			79 (91%)	45 (70%)	**0.001**
Low molecular weight heparin in pregnancy n (%)			20 (23%)	13 (20%)	0.69
Antihypertensives in pregnancy n (%)			6 (7%)	4 (6%)	1.00
Metformin and/or insulin in pregnancy n (%)			11 (13%)	9 (14%)	0.80
Cause of index stillbirth* n (%)					
		Placental	60 (69%)	47 (73%)	0.55
		Unexplained	26 (30%)	12 (19%)	0.12
		Other	1 (1%)	5 (8%)	0.08
Number of antenatal clinician reviews (median (IQR))			10 (8–13)	7 (5–12)	0.47
Number of fetal ultrasound scans (median (IQR))			9 (7–10)	9 (6–13)	0.65

BMI, body mass index; IQR, interquartile range; kg, kilograms; m^2^, meters squared; n, number; SD, standard deviation

*Placental causes of stillbirth included fetal growth restriction, hypertensive complications (pre-eclampsia) and histopathological findings on placental examination (e.g. maternal vascular malperfusion, fetal vascular malperfusion and fibrin deposition)

Women seen within the Rainbow Clinic were statistically less likely to spontaneously present in labor (OR, 0.05 [95% CI, 0.01–0.36]), however, more likely to undergo either a prelabor elective or emergency cesarean section (OR, 2.44 [95% CI, 1.20–4.94]) (Table 2). When the type of cesarean section was individualized, results were not statistically significant (*p* = 0.16 & *p* = 0.07 respectively). Furthermore, upon delineating the indication for either induction of labor or elective cesarean section, there were no significant differences in the frequencies of elective or medical indications between each cohort. There was a statistically significant advancement in the gestational age of delivery within the Rainbow Clinic group from a median delivery of 37 weeks and three days to 38 weeks (*p* = 0.004).

**Table 2:** Maternal outcomes for rainbow cohort and pre-Rainbow cohort

				Rainbow cohort (*n* = 87)	Pre-Rainbow Cohort (*n* = 64)	*P* Value
Hypertensive disease in pregnancy n (%)						
	Pre-eclampsia			4 (5%)	3 (5%)	1.00
	PIH			4 (5%)	4 (6%)	0.72
Onset of intrapartum care n (%)						
	Spontaneous labor			1 (1%)	13 (20%)	**< 0.001**
	IOL			47 (54%)	35 (55%)	0.94
		Elective IOL		30 (34%)	21 (33%)	0.83
		Medically indicated IOL		17 (20%)	14 (22%)	0.73
	Prelabor cesarean section			39 (45%)	16 (25%)	**0.01**
		Elective cesarean		25 (29%)	12 (19%)	0.16
			Malpresentation	1 (1%)	0 (0%)	1.00
			Previous cesarean	18 (21%)	7 (11%)	0.11
			Maternal request	6 (7%)	5 (8%)	1.00
		Emergency cesarean		14 (16%)	4 (6%)	0.07
Gestational age at birth (median (IQR))				38 + 0 (37 + 1–38 + 2)	37 + 3 (37 + 0–38 + 1)	**0.004**
Mode of delivery n (%)						
	Vaginal delivery			36 (41%)	37 (58%)	**0.046**
	Elective cesarean section			25 (39%)	12 (19%)	0.16
	Emergency cesarean section (prelabor and intrapartum)			26 (30%)	15 (23%)	0.38
Blood loss on delivery (ml; mean (SD)				512 (334)	692 (585)	**0.02**
PPH ≥ 500 ml n (%)				36 (41%)	29 (45%)	0.63
PPH ≥ 1000 ml n (%)				6 (7%)	15 (23%)	**0.004**
Requirement for transfusion n (%)				1 (1%)	1 (2%)	1.00
Postnatal maternal sepsis n (%)				3 (3%)	5 (8%)	0.28
Postnatal admission ≥ 5 days n (%)				2 (2%)	4 (6%)	0.40

IQR, interquartile range; IOL, induction of labor; ml, milliliters; n, number; PIH, pregnancy induced hypertension; PPH, post-partum hemorrhage; SD, standard deviation

Upon delivery fewer women within the Rainbow Clinic group delivered vaginally (*p* = 0.046). This is primarily due to a greater proportion of women undergoing a prelabor elective cesarean delivery and further women commencing on the induction of labor pathway, however finalizing with an emergency cesarean section during the intrapartum period. Despite a greater proportion of women within the Rainbow Clinic giving birth via cesarean section, there was a statistically significant reduction in mean blood loss at delivery (*p* = 0.02). This can be attributed to a statistically significant reduction in major postpartum hemorrhage occurrences ≥ 1000 ml (OR, 0.24 [95% CI, 0.09–0.67]). However, the requirement for postnatal transfusion was no different between groups (*p* = 1.00).

Perinatal outcomes are demonstrated within Table 3. 98% of pregnancies within the Rainbow Clinic group ended with a live birth compared with 92% within the pre-Rainbow Clinic group (*p* = 0.07). There were no cases of second trimester losses within the Rainbow Clinic group versus four cases within the pre-Rainbow Clinic group (*p* = 0.03). There was no statistical difference in the offspring birthweight, birthweight centile groups, rate of low birthweight and Apgar scores < 7 following birth between each cohort. Women within the Rainbow Clinic were statistically less likely to deliver a very or extremely premature infant (OR 0.17 [95% CI, 0.03–0.80] & OR 0.05 [95% CI, 0.00–0.93] respectively). There was a trend towards a reduction in NICU admission and length of stay within the Rainbow Clinic group, albeit not significant. The rate of composite adverse perinatal outcome was significantly less within the Rainbow Clinic versus the pre-Rainbow Clinic group (OR, 0.46 [95% CI, 0.22–0.98]).

**Table 3:** Perinatal outcomes for rainbow cohort and pre-Rainbow cohort

			Rainbow cohort (*n* = 87)	Pre-Rainbow Cohort (*n* = 64)	*P* Value
Outcome of pregnancy n (%)					
	Livebirth		85 (98%)	58 (92%)	0.07
	Stillbirth		2 (2%)	2 (3%)	1.00
	Second trimester miscarriage		0 (0%)	4 (5%)	**0.03**
Gender n (%)					
	Male		32 (37%)	40 (63%)	**< 0.001**
	Female		55 (63%)	21 (32%)	**< 0.001**
	Unknown		0 (0%)	3 (5%)	
Birthweight (grams; mean (SD))			2852 (529)	2904 (863)	0.66
Birthweight centile groups* n (%)					
	< 10th		18 (21%)	8 (13%)	0.19
	10th -90th		62 (71%)	45 (75%)	0.62
	> 90th		7 (8%)	7 (12%)	0.54
Low birthweight (< 2500 g) n (%)			18 (21%)	16 (25%)	0.53
Preterm birth n (%)					
		< 37 weeks	13 (15%)	15 (23%)	0.18
		< 32 weeks	2 (2%)	8 (13%)	**0.02**
		< 28 weeks	0 (0%)	6 (9%)	**0.005**
Apgar scores^					
		< 7 at 1 min	4 (5%)	4 (7%)	0.72
		< 7 at 5 min	3 (4%)	1 (2%)	0.65
Umbilical arterial cord pH (mean (SD))			7.22 (0.08)	7.24 (0.06)	0.36
Umbilical arterial cord pH < 7.05			0 (0%)	0(0%)	
Admission to NICU^ n (%)			13 (15%)	14 (24%)	0.18
Length of NICU admission (days; median (IQR))			7 (4–11)	9 (4–35)	0.10
Composite adverse perinatal outcome* n (%)			16 (18%)	21 (33%)	**0.04**

g, grams; IQR, interquartile range; n, number; NICU, neonatal intensive care unit; SD, standard deviation

*Results excluding the four cases of second trimester miscarriages within the pre-Rainbow group

^Results excluding cases of stillbirth and second trimester miscarriages within the two cohorts

Subgroup analysis of perinatal outcomes for cases in which the index stillbirth occurred secondary to a placental cause are shown in Table 4. 98% of the Rainbow Clinic group resulted in a livebirth versus 92% within the pre-Rainbow Clinic group (*p* = 0.10). There were no differences in mean birthweight, birthweight centile groups, gestational age at birth, rates of low birthweight and Apgar scores < 7. There was a trend towards a more advanced gestational age at delivery within the Rainbow Clinic, however, this was of borderline statistical significance (*p* = 0.05). There was a significant reduction in rates major postpartum hemorrhage occurrences (OR, 0.15 [95% CI, 0.04–0.58]) and extreme preterm delivery within the Rainbow Clinic group (*p* = 0.04). A trend in the reduction of NICU admission, length of stay and overall rates of composite adverse perinatal outcomes can be seen, however, results were not significant (*p* = 0.43, *p* = 0.12 and *p* = 0.16 respectively).

**Table 4:** Subgroup analysis of perinatal outcomes within the rainbow and pre-Rainbow cohorts whereby the index stillbirth was secondary to a placental cause

			Rainbow cohort (*n* = 60)	Pre-Rainbow Cohort (*n* = 47)	*P* Value
Outcome of pregnancy n (%)					
	Livebirth		59 (98%)	43 (92%)	0.10
	Stillbirth		1 (2%)	2 (4%)	0.58
	Second trimester miscarriage		0 (0%)	2 (4%)	0.19
Birthweight (grams; mean (SD))			2833 (507)	2813 (911)	0.89
Birthweight centile groups* n (%)					
	< 10th		12 (20%)	7 (15%)	0.49
	10th -90th		45 (75%)	35 (74%)	0.95
	> 90th		3 (5%)	5 (11%)	0.30
Low birthweight (< 2500 g) n (%)			13 (22%)	12 (26%)	0.64
Gestational age at birth (median (IQR))			37 + 5 (37 + 1–38 + 3)	37 + 3 (37 + 0–38 + 3)	0.05
PPH ≥ 1000 ml n (%)			3 (5%)	12 (26%)	**0.004**
Preterm birth n (%)					
		< 37 weeks	11 (18%)	11 (23%)	0.52
		< 32 weeks	2 (3%)	5 (11%)	0.24
		< 28 weeks	0 (0%)	4 (9%)	**0.04**
Apgar scores^					
		< 7 at 1 min	3 (5%)	4 (9%)	0.45
		< 7 at 5 min	1 (2%)	1 (2%)	1.00
Umbilical arterial cord pH (mean (SD))			7.21 (0.09)	7.25 (0.06)	0.10
Umbilical arterial cord pH < 7.05			0 (0%)	0(0%)	
Admission to NICU^ n (%)			10 (17%)	10 (21%)	0.43
Length of NICU admission (days; median (IQR))			7 (4–12)	19 (4–35)	0.12
Composite adverse perinatal outcome* n (%)			12 (20%)	15 (32%)	0.16

g, grams; IQR, interquartile range; n, number; NICU, neonatal intensive care unit; PPH, postpartum hemorrhage; SD, standard deviation

*Results excluding the two cases of second trimester miscarriages within the pre-Rainbow group

^Results excluding the single stillbirth case in the Rainbow group and the two cases of stillbirth and two cases of second trimester miscarriages within the pre-Rainbow group

Fifteen cases of women with singleton pregnancies and a prior history of perinatal loss were not seen within the Rainbow Clinic since its inception. Fourteen women (93%) were appropriately seen within specialist antenatal clinics. Thirteen women were seen within maternal medicine (five in diabetes clinic, three in cardiac clinic, two in maternal medicine clinic, two in endocrine clinic and one in epilepsy clinic), one woman was seen within the fetal medicine clinic and only one woman was seen within a general antenatal clinic rather than the Rainbow Clinic. Five women (33%) had a placental cause for stillbirth and four (27%) were unexplained. Seven cases (47%) resulted in cesarean section and eight cases resulted (53%) in vaginal delivery. Median gestational age at delivery was 37 weeks and 4 days versus 38 weeks and 0 days (*p* = 0.82). 27% incurred a blood loss ≥ 1000 ml versus 7% within the Rainbow Clinic group (OR 6.00 [95% CI, 1.42 − 25.34]. All cases resulted in livebirths, with three cases of preterm delivery < 37 weeks (*p* = 0.70), five cases of low birthweight (*p* = 0.32) and five admissions to NICU (*p* = 0.13). Composite adverse perinatal outcome was 30% versus 14% within the Rainbow Clinic group (*p* = 0.19).

## Discussion

This retrospective study aligns with the prior literature whereby a significant proportion (8%) of women with a history of stillbirth experienced a further stillbirth or second trimester miscarriage prior to the inception of the Rainbow Clinic [5, 15, 18, 19]. Following implementation of the Rainbow Clinic this reduced to 2%. Initiation of our specialist clinic resulted in a significant increase in gestational age at birth. Overall composite adverse perinatal outcome was significantly less frequent within the Rainbow Clinic, with significantly fewer cases of moderate and extreme prematurity. This occurred despite national data on preterm births demonstrating a slow increase year on year from 2020 [4]. There was an increased rate of prelabor cesarean section and a significant reduction in vaginal delivery. However, it should be noted that overall rates of cesarean section across the UK have been rising year by year since 2008; specifically increasing by 7% between 2017 and 2023 [20]. Despite this, there was a significant reduction in mean blood loss at delivery, including cases of major postpartum hemorrhage within the Rainbow Clinic group. However, rates of postnatal transfusion were no different, which may be of greater clinical relevance. Initial analysis of the Rainbow Clinic demonstrated a non-statistically significant trend towards a reduced rate of low birthweight, NICU admission and duration of stay. Finally, subgroup analysis of pregnancies whereby the cause of the index stillbirth was placental demonstrated a significant reduction in major postpartum hemorrhage and rates of extreme prematurity.

Consolidated evidence has demonstrated a significant increase in several adverse perinatal outcomes within subsequent pregnancies following an index stillbirth [5, 6, 18, 21]. Additionally, a recent analysis of 524 stillbirths highlighted that at least 10% of cases received inadequate antenatal care, increasing to 30% in those with a history of previous stillbirth, potentially contributing to these adverse outcomes [22]. This highlights a crucial need for clinicians to explore avenues to optimize care for these women. This concern was echoed as an urgent research priority by the UK stillbirth research priority setting partnership [23]. A possibility of achieving this may be the implementation of dedicated specialist antenatal services. However, currently there are only a few established UK clinics, thus leading to a paucity of literature examining the impact of these services on maternal-fetal outcomes. The lack of literature was clearly demonstrated by a Cochrane review in 2018 examining care of subsequent pregnancies following an index stillbirth, which included only 10 randomized control trials solely evaluating pharmacological treatments for these women rather than alternative interventions [24]. Consequently, much of the current limited research evaluating care models and outcomes is based on observational data alone. Abiola et al. investigated the impact of their Rainbow Clinic on clinical outcomes within 70 women, demonstrating a significant reduction in preterm birth, low birthweight at delivery, alongside a non-significant reduction in subsequent stillbirth and neonatal admission [15]. These results align closely with our findings and begin to demonstrate the positive clinical benefits these specialist clinics have on maternal-fetal outcomes.

Aside from the clinical implications associated with a previous history of stillbirth, it is vital to consider the psychological factors to both the women and the family. A recent systematic review of 144 studies examining the psychological impact following stillbirth highlighted a clear continuation of negative psychological symptoms within the next pregnancy [25]. Parents can display complex emotional responses which can be troublesome to address by staff within a busy routine antenatal clinic [12, 26]. Importantly, literature has noted that parents are comforted when clinicians demonstrate specific expertise within the area of subsequent stillbirth management [27]. Consequently, a focused multidisciplinary antenatal service devoted to this cohort of women is highly valuable to address such factors. Currently evidence of these specialist services has been well received by families, highlighting their concerns that were appropriately addressed, they felt listened to, whilst having an active role in their own care [10].

This study represents one of only a few studies evaluating the impact of a dedicated antenatal service for those with a prior history of stillbirth on clinical outcomes. The comprehensive in-depth analysis of maternal-fetal outcomes represents the main strength of this study. Additionally, only analyzing women within the pre-Rainbow Clinic whose clinical history would have made them eligible for Rainbow Clinic service ensures to optimize the comparisons made between each group, whilst minimizing selection bias. The small sample size and retrospective analysis represent the main weaknesses of the study, as this study is not powered to detect a difference in rare but serious outcomes. However, given the recent implementation of the Rainbow Clinic and the relative infrequency of perinatal mortality, a large cohort of patients was not expected to be acquired. Nevertheless, our study represents one of the largest cohorts of women to be analyzed. Importantly, we were able to demonstrate statistical significance within several crucial maternal-fetal outcomes, alongside a trend within others. Significance may be achieved with a larger cohort of patients, either from a single site or combining cohorts with other Rainbow Clinic services. Subsequent continued prospective data collection is vital for a thorough analysis of this specialist service. In addition, the retrospective nature of this study may be subject to unmeasured confounders, such as the Covid-19 pandemic which occurred during some of the years included within our pre-Rainbow group timeframe. This may have had an influence on maternal-fetal outcomes seen, particularly due to the consistency in care women may have received during this time. Further maternal confounding variables alongside changes in national clinical guidelines and implementation of national initiatives to improve perinatal outcomes during the data collection period may also have had an impact on the findings seen within this study. Given our relatively small sample size, adjustment for confounding variables would not be suitable. Therefore, awareness of potential confounding variables should be taken into consideration when interpreting the relationship between the independent and dependent variables presented. Finally, it would be pertinent to assess patient experience and attitudes towards the Rainbow Clinic service through qualitative studies, further improving the breadth of scope of this research.

## Conclusion

Stillbirth can have substantial clinical and psychological risks for the women and family within subsequent pregnancies. It is vital to discover and analyze avenues to improving the care women receive during subsequent pregnancies. This study demonstrates key insights into the beneficial impact of our Rainbow Clinic, a dedicated antenatal service for women with a history of stillbirth, on maternal-fetal clinical outcomes. Further prospective research, including qualitative analysis is necessitated to determine the true efficacy of these specialist clinics.

## Data Availability

The datasets generated and/or analyzed during the current study are not publicly available due to limitations of ethical approval involving the patient data and anonymity but are available from the corresponding author on reasonable request.

## Abbreviations

CI: confidence intervals
IQR: interquartile range
ml: milliliters
n: number
NICU: neonatal intensive care unit
OR: odds ratios
pH: potential of hydrogen
PPH: postpartum hemorrhage
SD: standard deviation
UK: United Kingdom
